# Role of verbal and non-verbal communication of health care providers in general satisfaction with birth care: a cross-sectional study in government health settings of Erbil City, Iraq

**DOI:** 10.1186/s12978-020-0894-3

**Published:** 2020-03-09

**Authors:** Hamdia Mirkhan Ahmed

**Affiliations:** College of Health Sciences, Center for Research and Education in Women’s Health, Hawler Medical University, Erbil, Iraq

**Keywords:** Communication, Personal communication, Women, Childbirth, Satisfaction

## Abstract

**Background:**

Effective communication by maternity care staff can help a woman during labor and birth have a positive birth experience. Due to limited knowledge regarding this topic in Iraqi Kurdistan, therefore, this study assessed: 1) The level of women’s satisfaction regarding verbal and non-verbal communication of midwives and physicians in the delivery room and 2) the association between this satisfaction level and socio-demographic and obstetric characteristics of the women and their general satisfaction with care during labor and delivery.

**Methods:**

A cross-sectional study was conducted on a convenient sample of 1196 women recruited between January and March 2019 from Erbil city, Iraq, who gave birth in the year before that. Data were collected from women through direct interview. A questionnaire which included sociodemographic, obstetrical information and 28 items related to verbal and non-verbal communication of physicians and midwives in the delivery room was used. Chi-square tests were used to find the association between dependent and independent variables.

**Results:**

Although 58.4% of the women were generally satisfied with communication of midwives and physicians in the delivery room, a large percentage (41.6%) were not satisfied. Only 14.6 and 27.3% of the women were completely satisfied with verbal and non-verbal communication of health care providers, respectively. There was a statistically significant association between women’s satisfaction with care during labor and their satisfaction with health care providers’ communication; 70.4% of women who were satisfied with care during birth were also satisfied with the communication of delivery room staff. There were statistically significant associations between the satisfaction of women with the communication of midwives and physicians and their level of education, parity, having stillbirth or neonatal death, and the setting of the last delivery.

**Conclusions:**

Women’s satisfaction with verbal and nonverbal communication of health care providers in the delivery room is associated with their satisfaction with birth care. Improving communication skills of health care providers can be a considerable part of improving care in delivery room.

## Plain English summary

Effective communication by maternity care staff is an important factor for women’s satisfaction with care during labor and delivery. This study assessed women’s satisfaction with personal communication from health care providers including physicians and midwives in the delivery room. 1196 women participated. Results show that a relatively small percent of women were completely satisfied with the communication, although more than half of the women were generally satisfied with the communication. Seventy point 4 % of those women who were satisfied with care during birth were also satisfied with communication of the delivery room staff. We concluded that women’s satisfaction with verbal and nonverbal communication of health care providers in the delivery room is associated with their satisfaction with birth care.

## Background

Delivery is one of the most important events in a woman’s life, and one in which mental and physical stress is unavoidable [1]. This event is recognized as having deep mental, social, and emotional aspects that remain in the mother’s consciousness forever; therefore, unpleasant events during delivery can have a negative long-term mental effect [2, 3].

During parturition, mothers need attention, understanding, empathy, guidance, and support in addition to clinical care [4]. They recognized the potential vulnerability of themselves and their baby through the process, and the essential uncertainty about what might happen. This was associated with a strong desire for safe, supportive, kind, respectful and responsive care during labor and birth. These characteristics applied to birth companions, professional and lay care givers, and to the processes and environment of care. Attitudes and behaviors of staff, the quality of the relationship between women and care providers, and the resources and atmosphere of the local facility has effect on women’s experience [5]. Relevant studies show that a pivotal factor for a positive delivery experience is the comprehensive support of midwives. On the other hand, weak midwife communication skills could potentially have deleterious effects on physical, mental, social, and economical aspects of healthcare [1].

Effective communication by maternity care staff can help a woman during labor and birth to have a positive birth experience [6]. Childbirth can be a frightening experience for many women, but it should also be a joyous occasion and every woman should feel valued, respected, and appreciated by those who aid her in her journey of bringing new life into the world [7]. Effective communication between maternity care providers and women in labor, using simple and culturally acceptable methods, is recommended by the World Health Organization [8]. Therefore, enhancement of physicians’ and midwives’ communication skills is of key importance to promote the health of expecting mothers [9].

Studies performed in maternity wards showed that appropriate communication could be the main determining factor for satisfaction of mothers with the provided care [10]. An effective relationship between midwives and mothers can result in enhanced satisfaction, lowered blood pressure, anxiety, and pain, an increased sense of security, mutual trust, and interaction, an improved ability to make an informed decision, a reduced fear of a vaginal birth, an enhanced sense of assurance, better control of the delivery process, and most importantly, improved health [1].

Patient satisfaction is an important and commonly used indicator for measuring the quality of health care. Patient satisfaction affects clinical outcomes, patient retention, and medical malpractice claims. It affects the timely, efficient, and patient-centered delivery of quality health care. Patient satisfaction is thus a proxy but a very effective indicator to measure the success of doctors and hospitals [11].

Studies show that younger women, women with a low education level, rural residents and primiparas reported discrimination and unfriendly behaviors by providers and staff in the delivery room. As a result, they were not satisfied with their labor care [12–14]. No research could find on this subject in the Kurdistan region of Iraq; therefore, I investigated midwives’ and physicians’ communication skills in the delivery room in Erbil city, Iraq. This study assessed 1) women’s perception about the quality of rapport between them and their providers in the delivery room; 2) the association between this satisfaction and sociodemographic characteristics of the women; and 3) the association between women’s satisfaction and their general satisfaction with care during labor and delivery.

## Methods

### Study population

A cross-sectional study was conducted in Erbil city, in the Kurdistan region of Iraq during the period of January 20, 2019 to March 15, 2019. The inclusion criteria were having at least one birth experience (a vaginal delivery) within the past year, either in Rezgary hospital, a Maternity Teaching Hospital or Malafandy, a primary health center in Erbil city. Maternity Teaching Hospital is the biggest specialized public hospital for women’s Health care especially labor and delivery care with 300 hundred beds and 30 beds in delivery room. Rezgary Hospital is a general hospital including a unit for women’s health care services with capacity of 6 beds and Malafandy Primary Health Center has a small unit with capacity of 5 beds for labor and delivery care of women with low risk and normal health conditions. All of these institutions are public health settings. Women who delivered in a private hospital, had a psychological problem or were not interested in participating were excluded. Through convenient sampling, 1500 women were approached to participate in the study; 1234 met the inclusion criteria and 1196 women agreed to participate. These women were either accompanying another person to the hospital or health center, or coming to immunize one of their children.

### Data collection

Data were collected through direct interview after we received informed consent. The purpose of the study was explained to the women and they were assured that all personal information would remain confidential. Three midwifery students helped the researcher with data collection after they were trained on how to interview women and collect the data.

A questionnaire was developed by the author after a massive review of literature which included two main parts: sociodemographic and obstetrical characteristics such as age, educational level, occupation, residency, parity, history of abortion, sex of the baby, admission of the baby to the ICU and 28 items related to verbal (16 items) and non-verbal (12 items) communication of physicians and midwives in delivery room [13–18].

#### Outcome variables

Responses to 28 items about the verbal and non-verbal communication of providers were scored as follows: a response of not satisfied was awarded 1 point, a response of don’t know was awarded 2 points, and a response of satisfied was awarded 3 points. After obtaining the overall satisfaction of women, we summarized the results into two groups: satisfied and unsatisfied including both verbal and non-verbal communication items. Those who were not satisfied and “don’t know” were merged together as don’t know indirectly implied that women were not satisfied. Only a small percentage of the sample had allocated themselves to the “don’t know” group. Those with a score of less than 56 (the midpoint between the minimum 28 and maximum 84) were considered as not satisfied and those with a score 57 to 84 were considered satisfied. Separately for verbal and non-verbal satisfaction, overall satisfaction was divided into four groups. For verbal communication items: completely not satisfied (those who received a total score of 16 points on the questionnaire, corresponding to not satisfied on every question), partly not satisfied (those who received a score between 17 and 32 points), partially satisfied (a total score of 33–47 points) and completely satisfied (a total score of 48 points, indicating complete satisfaction on every one of the 16 items). For non-verbal communication items, the total score for the corresponding four categories were: 12 points, 13–24 points, 25–35 points, and 36 points, respectively.

A separate question was asked regarding the general satisfaction with care during labor with three response of yes, no or partially.

#### Exposure variables

Exposure variables were age, educational level, occupation, residency, parity, having an abortion and stillbirth or neonatal death, the sex of the baby, admission of the baby to the ICU, having an episiotomy, and place of last delivery (a maternity hospital, Rezgary Hospital or Malafandi Center). The baby’s sex, admission to the ICU, having an episiotomy and place of delivery described the mother’s most recent delivery. Age was grouped as less than 20 years (which was considered an adolescent), 20 to 29, 30 to 39, and 40 or more years old. For educational level, basic means finishing 9 years of education, secondary means completing 12 years of education, and institute and BSc includes those who finished 2 years or more at a university. Residency is divided as urban which is Erbil city, suburban which includes towns within a half hour to one hour’s drive from Erbil city, and rural, including towns and villages beyond the suburban areas. As most Kurdish women are housewives, occupation was grouped as housewife, employed or students. Parity was grouped as primipara (had one delivery), multipara (2 to 4 deliveries) and grand multipara (5 and more deliveries).

### Statistical analysis

Data entered into and analyzed by SPSS program (Version 21) including frequency, percentage and chi-square test. Sociodemographic and obstetric data, satisfaction with verbal and nonverbal communication items and overall satisfaction were summarized by frequency and percentage. A chi-square test was used to find the association of general satisfaction with sociodemographic and obstetric variables. .

The study was approved by the Scientific Committee of the College of Nursing/Hawler Medical University.

## Results

The sociodemographic characteristics of the study sample were as follows: almost half of the study sample was aged between 20 and 29 years old, The highest percentage (40.8%) graduated from basic school, more than half (54.4%) were from an urban area, and the majority of participants (92.8%) were housewives. The highest percentage (62.5%) of the women were multigravida, 34.2% had a history of abortion, 91.2% had no history of stillbirth or neonatal death, 63.6% experienced episiotomy or laceration during their most recent delivery and only 13.1% of their babies were admitted to the hospital NICU after delivery (Table 1).

**Table 1:** Sociodemographic and obstetric characteristics of the study subjects (*N* = 1196, Erbil city, Iraq, 2019)

Variables	Number	Percent
**Age group (years)**		
- less than 20	93	7.8
- 20–29	597	49.9
- 30–39	445	37.2
- 40 or more	61	5.1
**Education level**		
- illiterate	268	22.4
- read and write	168	14.0
- basic	488	40.8
- secondary	139	11.7
- institute /BSc^a^	133	11.1
**Residency**		
- urban	651	54.4
- suburban	340	28.4
- rural	205	17.1
**Occupation**		
- housewife	1110	92.8
- employed	59	4.9
- student	27	2.3
**Parity**		
- primipara	238	19.9
- multipara	748	62.5
- grand multipara	210	17.6
**Number of abortions**		
- no abortion	787	65.8
- 1–2	356	29.8
- 3 and more	53	4.4
**Number of stillbirths**		
- 0	1091	91.2
- 1	88	7.4
- 2–3	17	1.4
**Episiotomy & laceration (in most recent delivery)**		
- yes	761	63.6
- no	435	36.4
**Sex of baby**		
- male	589	49.2
- female	607	50.8
**Admission to NICU**^**b**^		
- yes	157	13.1
- no	1039	86.9

^a^*BSc* Baccalurius degree, ^b^*NICU* Neonatal Intensive Care Unit

Table 2 shows the satisfaction of participants with verbal and non-verbal communication of midwives and physicians during their labor and delivery. More than half of the study sample were not satisfied with this verbal communication: “introduce self” (66.5%), “greeted the patient” (61.7%), “checked the patient’s understanding” (58%), “asked whether patient had other issues or concerns” (50.2%). Regarding non-verbal communication around two third of the women were satisfied with 11items from 12, described in Table 2 . The highest percentage (43.7%) of the women not satisfied was for the item “maintain a patient’s privacy during the physical examination.”

**Table 2:** Participant satisfaction with verbal and nonverbal communication of the midwives and physicians during their labor and delivery (*N* = 1196, Erbil city, 2019)

Verbal communication	Not Satisfied		Don’t know		Satisfied	
The provider…	No	%	No	%	No	%
greeted the patient	738	61.7	22	1.8	436	36.5
offered a seat to the patient	461	38.5	25	2.1	710	59.4
introduced self to the patient	795	66.5	38	3.2	363	30.4
used the patient’s name	448	37.5	36	3	712	59.5
used open questions to encourage the patient to freely provide information about her concerns	385	32.2	42	3.5	769	64.3
encouraged the patient to speak as long as she needed to convey what she considered important	363	30.4	55	4.6	778	65.1
verbally confirmed that they would respond to the patient’s request	342	28.6	92	7.7	762	63.7
carefully listened to the patient	283	23.7	82	6.9	831	69.5
spoke in a calm and gentle manner	269	22.5	68	5.7	859	71.8
used simple language that was easy to understand	266	22.2	69	5.8	861	72
explained the examination and assessment	547	45.7	94	7.9	555	46.4
explained patient’s health problems and diagnosis	573	47.9	103	8.6	520	43.5
discussed treatment options and offered choices	507	42.4	105	8.8	584	48.8
provided information tailored to the patient’s problems and concerns	537	44.9	95	7.9	564	47.2
checked the patient’s understanding of information	693	58	101	8.4	402	33.6
asked whether the patient had other issues or concerns she would like to discuss	600	50.2	105	8.8	491	41.1
**Nonverbal communication**						
had a neat and tidy personal appearance	304	25.4	168	14	724	60.5
made the patient feel welcome	415	34.7	74	6.2	707	59.1
displayed patience when visiting with the patient	320	26.8	82	6.9	794	66.4
maintained good eye contact with the patient	323	27	95	7.9	778	65.1
used facial expressions to convey interest and attention	312	26.1	97	8.1	787	65.8
used a caring and attentive body posture when approaching the patient	300	25.1	87	7.3	809	67.6
used a professional tone of voice that also conveyed empathy and caring	306	25.6	104	8.7	786	65.7
listened carefully and not interrupt the patient while speaking	315	26.3	62	5.2	819	68.5
used nonverbal cues while listening to show attentiveness and empathy	337	28.2	71	5.9	788	65.9
encouraged the patient to keep talking with posture and facial expressions	333	27.8	68	5.7	795	66.5
maintain a patient’s privacy during the physical examination	522	43.7	81	6.8	593	49.6
visit patients individually rather than grouping them together	390	32.6	100	8.4	706	59

Although 58.4% of the women were generally satisfied with the communication of midwives and physicians in the delivery room, a large percentage (41.6%) were not satisfied. Only 14.6 and 27.3% of the women were completely satisfied with verbal and non-verbal communication of their health care providers, respectively (Table 3).

**Table 3:** Overall satisfaction of women with communication of midwives and physicians during their labor and delivery (N = 1196, Erbil city, 2019)

Level of satisfaction with communication	No. (%)
**Overall satisfaction**	
- not satisfied	497 (41.6)
- satisfied	699 (58.4)
**Overall verbal communication**	
- completely not satisfied	51 (4.3)
- partially not satisfied	477 (39.9)
- partially satisfied	493 (41.2)
- completely satisfied	175 (14.6)
**Overall non-verbal communication**	
- completely not satisfied	42 (3.5)
- partially not satisfied	402 (33.6)
- partially satisfied	426 (35.6)
- completely satisfied	326 (27.3)

As Table 4 shows, there were statistically significant associations between the satisfaction of women with the communication of their midwives and physicians and the women’s level of education (*p* = 0.021), parity (*p* = 0.002), and having a stillbirth (*p* = 0.023), as well as a highly significant association with the place of their last delivery (p = < 0.001). The same table shows that there was a statistically significant association between women’s satisfaction with their care during labor and their satisfaction with their health care providers’ communication (*p* = < 0.001). Seventy point 4 % of those women who were satisfied with care during birth were also satisfied with the communication of the midwives and physicians in the delivery room.

**Table 4:** Association between sociodemographic, obstetric characteristics and birth outcome satisfaction with overall communication satisfaction (*N* = 1196, Erbil city, 2019)

Variables	Overall satisfaction with provider communication		***P***-value
	Not satisfied No(%)	Satisfied No(%)	
Age group			
- less than 20	35 (37.6)	58 (62.4)	
- 20–29	264 (44.2)	333 (55.8)	0.066
- 30–39	181 (40.7)	264 (59.3)	
- 40 and more	17 (27.9)	44 (72.1)	
Level of education			
- illiterate	103 (38.4)	165 (61.6)	
- read and write	74 (44)	94 (56)	0.021
- basic	185 (37.9)	303 (62.1)	
- secondary	68 (48.9)	71 (51.1)	
- institute /BSc	67 (50.4)	66 (49.6)	
Occupation			
- housewife	460 (41.4)	650 (58.6)	0.944
- employed	25 (42.4)	34 (58.6)	
- student	12 (44.4)	15 (55.6)	
Residency			
- urban	261 (40.1)	390 (59.9)	0.230
- suburban	140 (41.2)	200 (58.8)	
- rural area	96 (46.8)	109 (53.2)	
Parity			
- primipara	120 (50.4)	118 (49.6)	0.002
- multipara	284 (38)	464 (62)	
- grand multi para	93 (44.3)	117 (55.7)	
Number of stillbirths			
- none	461 (42.3)	630 (57.7)	0.023
- one	26 (29.5)	62 (70.5)	
- two to three	10 (58.8)	7 (41.2)	
Number of abortions			
- none	336 (42.7)	451 (57.3)	0.492
- one to two	106 (40.2)	158 (59.8)	
- three and more	55 (37.9)	90 (62.1)	
Admission to NICU^a^			
- yes	74 (47.1)	83 (52.9)	0.128
- no	423 (40.7)	616 (50.3)	
Sex of baby			
- male	247 (41.9)	342 (58.1)	0.793
- female	250 (41.2)	357 (58.8)	
Place of last delivery			
- a maternity hospital	429 (39.1)	667 (60.9)	< 0.001
- Rezgary Hospital	66 (71.7)	26 (28.3)	
- Malafandi Center	2 (25)	6 (75)	
Had episiotomy			
- yes	324 (42.6)	437 (58.4)	0.344
- no	173 (39.8)	262 (60.2)	
Generally satisfied with care during birth			
- yes	276 (29.6)	658 (70.4)	< 0.001
- no	177 (93.7)	12 (6.3)	
- partially	44 (60.3)	29 (39.7)	

^a^*NICU* neonatal intensive care unit

## Discussion

In the present study, 1196 women were examined to find out their satisfaction with the communication of physicians and midwives during their labor and delivery and its relation with their overall satisfaction with received related care. The results show that the majority of women who were satisfied with their care were also satisfied with the communication of their health care providers. Only 14.6 and 27.3% of women were completely satisfied with all verbal and nonverbal communication items measured, respectively. Satisfaction with this communication was greater among women who were multigravida and grand multipara. As the women’s education level increased, their satisfaction with the communication decreased which may due to this fact that educated women have a better understanding of the kind care possible given by health care providers. Age was not a significant factor in the satisfaction of women with communication.

The results of the present study regarding the association of satisfaction with sociodemographic characteristics of the women are in agreement with the results of many other studies. For instance, the results of a study done on 790 Australian women shows that parity, level of school-based education and place of residence were associated with differences in women’s overall ratings of care; however, age was not associated with ratings of care [19]. In a 2016 study, labor observations and 2672 surveys in Ghana, Guinea, Myanmar, and Nigeria, age was predominantly the single factor associated with different types of mistreatment. Younger women (15–19 years) were more likely to experience any physical abuse, verbal abuse, or stigma or discrimination, after adjusting for country, education, marital status, and parity. Younger women with no education and younger women with some education were more likely to experience verbal abuse, compared with older women (≥30 years) [14]. In a cross-sectional study by Okafor et al. in Enugu State in Nigeria, 20% of women reported discrimination on the basis of ethnicity, low social class, young age and HIV seropositive status, however, in four cross-sectional studies, women of low socioeconomic status and with no formal education reported experiences of unfriendly and harsh attitudes of staff in higher proportions [12].

Women in labor have a need for companionship, empathy and help, and descriptive studies of women’s childbirth experiences have suggested four dimensions to the support that they want during labor: emotional support, informational support, physical support and advocacy [20]. Both the emotional and informational support depend on the communication skills of physicians and midwives.

The results of the present study are similar to results of other studies done in different countries which also show the importance of women’s satisfaction with communication as an important factor in the general satisfaction of women’s birth care [20–22]. In a descriptive study from Iran, 50 midwives were evaluated by parturient women. The majority of them evaluated midwives’ communicative behaviors at an acceptable level [1].

In an institution-based cross-sectional descriptive study from Ethiopia, a total of 423 postpartum women were interviewed to assess their satisfaction in a maternal health care setting. The results show that the proportion of mothers who were completely satisfied with the health care they received ranged between 2.4 to 21%. The provider’s communication with their clients yielded complete client satisfaction rates ranging between 0.7 to 26% [16].

Not being satisfied with provider communication was a reason for being dissatisfied with overall care among 400 women who delivered in a hospital in Egypt [23].

Results of a cross-sectional survey on 1004 women in India indicated that improving interpersonal interaction with nurse-midwives, and ensuring privacy during childbirth and the hospital stay are recommended first steps to improve women’s childbirth satisfaction [15].

In a qualitative study from Nigeria, the quality of communication of health care providers in a delivery room was recognized as an important factor in improving women’s experience of childbirth [24].

However, an increasing body of work over the last 20 years has demonstrated the relationship between doctors’ non-verbal communication (in the form of eye-contact, head nods and gestures, position and tone of voice) with the following outcomes: patient satisfaction, patient understanding, physician detection of emotional distress, and physician malpractice claim history. Although more work needs to be done, there is now significant evidence that doctors need to pay considerable attention to their own non-verbal behavior [25]. Conversely, nonverbal dominance, in the form of long physician speaking time or a dominant tone of voice, for instance, has a negative impact on satisfaction [26]. Also, it has been shown that a physician’s nonverbal behavior that expresses concern, for instance, through frequent eye contact, a concerned facial expression, or close interpersonal distance, leads to more patient trust than a physician’s behavior that conveys more distance. Regarding patient adherence, it has been shown that physician touching of the patient increases patient adherence with their medication [26].

In a qualitative study on 16 mothers in Iran, results show that outcomes of a positive midwife-motherrelationship in the delivery room can lead to facilitation of childbirth, a positive birth experience, mental health promotion and improvement in the mother’s quality of life. An effective midwife-mother relationship can lead to positive childbirth outcomes and promotion of maternal and neonatal health [21].

In a mixed-methods systematic review, a lack of evidence on the impact of interventions to support effective communication between maternity care staff and healthy women during labor and birth were identified [27]. A study from Syria reported that a communication skills training intervention for resident doctors were not associated with higher satisfaction reported by women. In the context of a highly crowded and stressful environment where middle-class and low-class Syrian women deliver, a specially-designed training package in interpersonal and communication skills for residents did not achieve an overall improvement in women’s satisfaction with the doctor– woman relationship in labor and delivery rooms. However, certain items in the doctors’ behavior have improved. It would be worth investigating whether the package would improve women’s satisfaction in less stressful settings, but also it is worth looking at other possible interventions in maternity care practice such as doctor–midwife collaboration or attendance of birth companions in such settings. Despite the lack of evidence from these studies, the need to improve interpersonal skills of medical doctors and obstetricians specifically should be reinforced, as good communication is central to quality healthcare [28].

Verbal and nonverbal communication of health care providers in the delivery room have a significant impact on women’s satisfaction with care, but we have to consider others factors, too. WHO recommends effective communication between maternity care providers and women in labor, however during the guideline development process, the Guideline Development Group (GDG) identified important knowledge gaps that need to be addressed through primary research regarding the effects of communication skills training on women’s and providers’ experiences, level, type and other characteristics of communication and optimal skilled birth attendant to-woman ratio for delivery of effective communication [8]. Further studies are needed to more precisely identify the role of communication in women’s satisfaction during labor and delivery by considering all aspects of care.

### Strengths and limitations

This study included a large sample size. Additionally, studying verbal and non-verbal communication was a strength of the present study, whereas the convenient sampling approach and limiting our sample to include only the experiences of births in public hospitals were limitations.

## Conclusions

The overall satisfaction of women with the communication of their health care providers in the delivery room was 58.4%. A relatively small proportion (14.6, 27.3%) of women were completely satisfied with verbal and non-verbal communication of health care providers in the delivery room, respectively. The majority of women who were generally satisfied with their birth care were also satisfied with the communication of physicians and midwives during birth. The results of the present study have clarified one necessary aspect for quality improvement in intrapartum care in maternity hospitals of the Kurdistan region of Iraq. Health policy makers and stakeholders can play an important role in developing strategies for improving communication skills of health care providers.

## Data Availability

The datasets used and/or analyzed during the current study are available from the corresponding author on reasonable request.

## Abbreviations

WHO: World Health Organization
NICU: Neonatal Intensive Care Unit
